# Pedicle Ligation Prior to Milligan-Morgan Hemorrhoidectomy for Advanced Grade IV Hemorrhoids: An Experience From a Specialized Proctology Unit

**DOI:** 10.7759/cureus.103075

**Published:** 2026-02-06

**Authors:** Abdelilah Hamada, Achraf Bahi, Mohamed Amine Benhaddi, Hicham Laraqui

**Affiliations:** 1 Visceral Surgery, Hôpital Militaire d'Instruction Mohammed V (HMIMV), Rabat, MAR; 2 Proctology, Hôpital Militaire d'Instruction Mohammed V (HMIMV), Rabat, MAR

**Keywords:** hemorrhoidal vascular control, intraoperative bleeding, milligan-morgan hemorrhoidectomy, operative blood loss, pedicle ligation, surgical safety, surgical strategy

## Abstract

Grade IV hemorrhoidal disease represents the most advanced form of hemorrhoids and is frequently associated with severe prolapse, chronic inflammation, and a high risk of bleeding. Although minimally invasive techniques have gained popularity, excisional hemorrhoidectomy remains the most effective treatment for advanced disease. We report a case highlighting the role of pedicle ligation prior to Milligan-Morgan hemorrhoidectomy in improving surgical safety.

A 32-year-old man with no significant medical history presented with symptomatic, irreducible grade IV hemorrhoids refractory to conservative treatment. Open excisional hemorrhoidectomy using the Milligan-Morgan technique was performed following the systematic ligation of the hemorrhoidal pedicles. The procedure was completed without intraoperative complications. Postoperative recovery was uneventful, with excellent clinical and functional outcomes.

In advanced hemorrhoidal disease, the risk of intraoperative bleeding is increased due to marked vascular congestion and chronic inflammation. Pedicle ligation prior to excision may enhance hemostasis, improve operative field exposure, and reduce perioperative complications. This strategy is particularly relevant in grade IV hemorrhoids, where minimally invasive techniques have shown higher recurrence rates.

Pedicle ligation before Milligan-Morgan hemorrhoidectomy is a safe and effective strategy in the management of grade IV hemorrhoids and may improve surgical outcomes in selected patients.

## Introduction

Hemorrhoidal disease is a prevalent anorectal condition affecting up to 40% of adults during their lifetime [1]. While most patients present with early-stage disease amenable to conservative or office-based treatment, advanced hemorrhoidal disease continues to pose a significant therapeutic challenge.

According to Goligher's classification, grade IV hemorrhoids are permanently prolapsed and irreducible, often associated with mucosal ulceration, thrombosis, and chronic inflammation. These features contribute to an increased risk of bleeding and postoperative complications [2].

Despite the development of minimally invasive techniques, their role in grade IV hemorrhoids remains controversial. Conventional excisional hemorrhoidectomy continues to be recommended as the definitive treatment for advanced disease [3-5].

In advanced grade IV hemorrhoids, marked vascular congestion and chronic inflammatory changes increase the risk of intraoperative bleeding, highlighting the potential value of pedicle ligation to improve hemostasis during excisional surgery.

The Milligan-Morgan hemorrhoidectomy remains a widely accepted technique. We report a case emphasizing the benefit of systematic pedicle ligation prior to excision as a strategy to improve hemostasis and surgical safety in severe hemorrhoidal disease [6].

## Case presentation

A 32-year-old man presented with a several-month history of painful anal prolapse, recurrent rectal bleeding, and discomfort during defecation. Symptoms were persistent and refractory to dietary modification and medical therapy.

**Figure 1:**
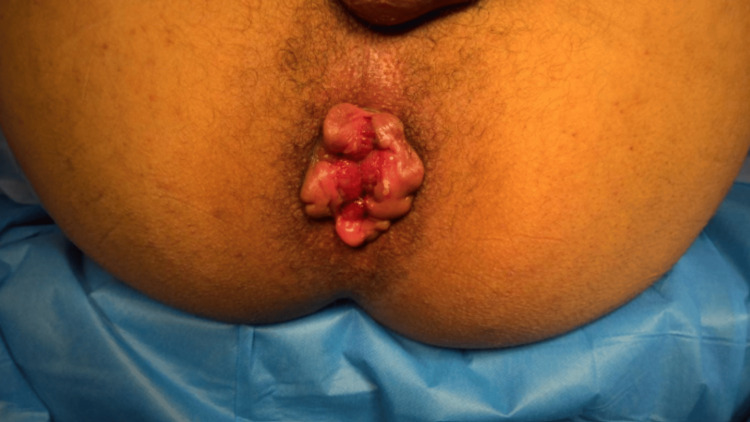
Preoperative view demonstrating large, permanently prolapsed grade IV hemorrhoids with marked congestion and inflammatory changes

The patient had no significant past medical history and no previous anorectal surgery. Clinical examination revealed large, edematous, permanently prolapsed hemorrhoidal cushions consistent with grade IV hemorrhoids, associated with marked mucosal congestion and inflammation (Figure 1).

Routine laboratory investigations were normal. After a detailed discussion of treatment options, surgical management was recommended, and informed consent was obtained.

Surgical technique

The procedure was performed with the patient in the lithotomy position under regional anesthesia. After appropriate exposure, the hemorrhoidal cushions were identified.

Pedicle identification was achieved through standard anatomical recognition using gentle traction on the hemorrhoidal pile mass, without the use of Doppler guidance.

Given the marked vascular congestion and inflammatory changes, systematic ligation of the hemorrhoidal pedicles was first performed using absorbable sutures to control arterial inflow. This step resulted in an immediate reduction of bleeding and improved visualization.

Subsequently, open excisional hemorrhoidectomy was carried out using the Milligan-Morgan technique, with excision of the three principal hemorrhoidal cushions while preserving adequate mucocutaneous bridges to minimize the risk of anal stenosis.

**Figure 2:**
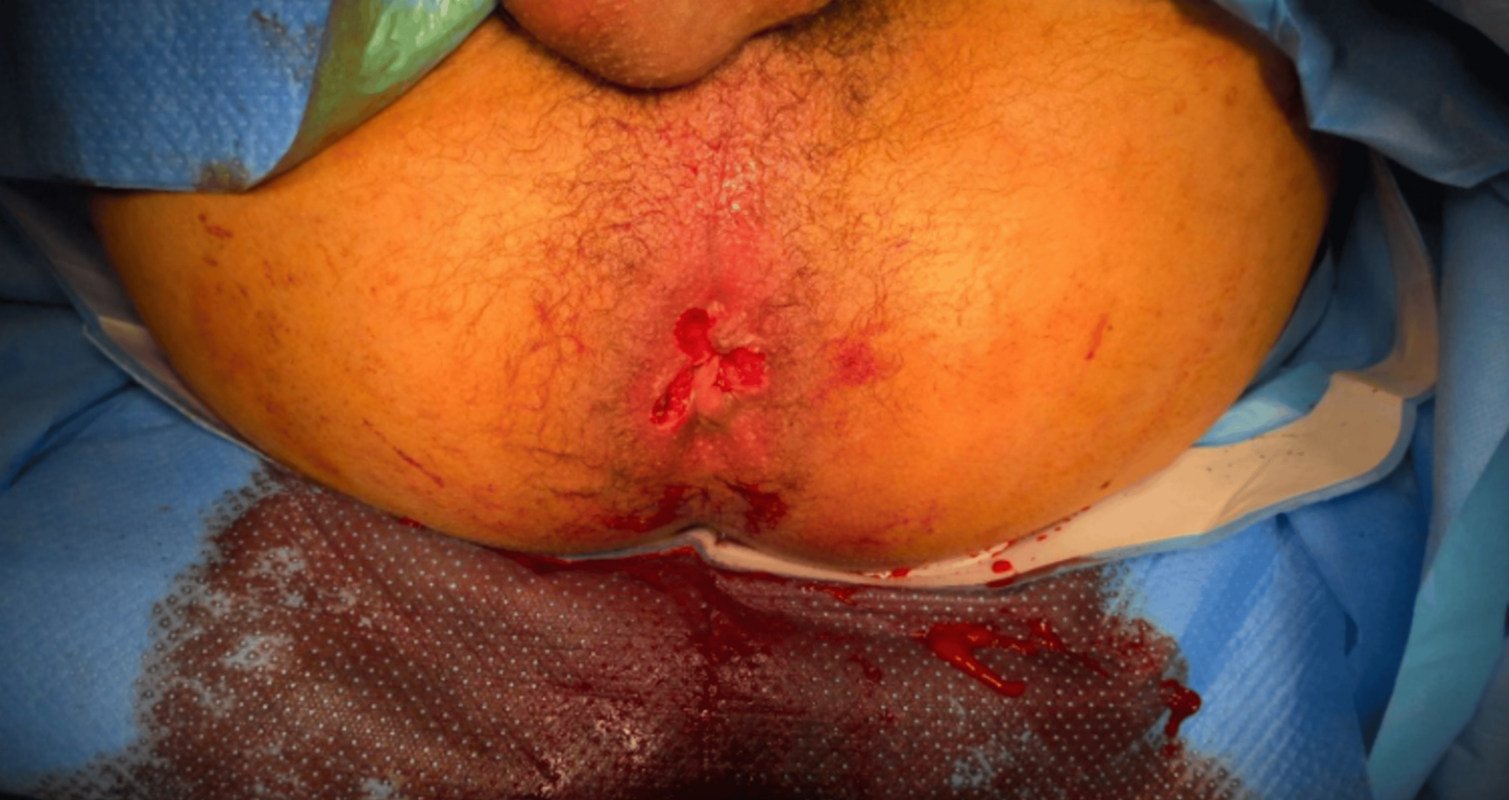
Immediate postoperative appearance following Milligan-Morgan hemorrhoidectomy after pedicle ligation, showing satisfactory excision and effective hemostasis

Meticulous hemostasis was achieved. No intraoperative complications occurred (Figure 2).

Postoperative outcome

Postoperative care included oral analgesia, stool softeners, and local wound management. Pain was moderate and well-controlled.

No postoperative bleeding, infection, urinary retention, or anal stenosis was observed. The patient reported complete resolution of symptoms and normal bowel function at follow-up, with no recurrence.

## Discussion

Management of grade IV hemorrhoidal disease remains controversial. While minimally invasive techniques may reduce postoperative pain, multiple studies have demonstrated higher recurrence rates and reduced efficacy in advanced disease compared with excisional hemorrhoidectomy [3-5].

Excisional hemorrhoidectomy remains the gold standard for grade IV hemorrhoids, providing superior long-term symptom control. However, intraoperative bleeding remains a major concern due to increased vascularity and chronic inflammatory changes [1,4].

In the present case, preoperative findings of marked congestion and mucosal inflammation supported the decision to perform pedicle ligation prior to excision. This approach facilitated improved hemostasis, better operative field exposure, and an uncomplicated postoperative course, in line with classical anatomical principles of hemorrhoidectomy [6].

## Conclusions

Pedicle ligation prior to Milligan-Morgan hemorrhoidectomy is a valuable surgical strategy in advanced grade IV hemorrhoidal disease. This approach may improve hemostasis, enhance operative safety, and contribute to excellent clinical outcomes. Conventional excisional hemorrhoidectomy remains an essential technique in the modern management of severe hemorrhoids.

However, these observations are based on a single-case experience, and further comparative studies are required to confirm the reproducibility and broader clinical impact of this approach.
